# A Comparative Study on Absorption of Gaseous Formaldehyde by Electrospun Biomass Carbon Nanofiber Membranes Modified by Plasma Activation and Chemical Treatment

**DOI:** 10.3390/molecules30102184

**Published:** 2025-05-16

**Authors:** Qian He, Jinhui Xiong, Huanbo Wang, Linkun Xie, Xijuan Chai, Lianpeng Zhang, Siqun Wang, Guanben Du, Kaimeng Xu

**Affiliations:** 1 Yunnan Provincial Key Laboratory of Wood Adhesives and Glued Products, International Joint Research Center for Biomass Materials, College of Materials and Chemical Engineering, Southwest Forestry University, Kunming 650224, China; qianh0516@163.com (Q.H.); xiongjinhui513@163.com (J.X.); xielinkun@163.com (L.X.); lpz@zju.edu.cn (L.Z.); guanben@swfu.edu.cn (G.D.); 2 Center for Renewable Carbon, The University of Tennessee, Knoxville, TN 37996, USA; swang@utk.edu

**Keywords:** gaseous formaldehyde, adsorption, electrospinning, carbonization, chemical treatment, cold plasma activation

## Abstract

To comparatively study the effects of cold plasma activation and chemical treatment on the adsorption capacities of biomass carbon nanofiber membranes (BCNMs), microcrystalline cellulose (MCC) and chitosan (CS) were used to fabricate porous BCNMs by electrospinning and carbonization. Two modification methods, including oxygen (O_2_) plasma activation and chemical treatment using nitric acid (HNO_3_), sulfuric acid (H_2_SO_4_), hydrogen peroxide (H_2_O_2_), and urea, were further employed to enhance their adsorption performance. Various carbonyl group (C=O), ether bond (C-O), carboxyl group (O-C=O) and pyridinic nitrogen (N), pyrrolic N, and quaternary N functional groups were successfully introduced onto the surface of the BCNMs by the two methods. The BCNM-O_2_ showed optimal formaldehyde absorption capacity (120.67 mg g^−1^), corresponding to its highest contents of N, O-containing functional groups, and intact network structure. However, chemical treatment in strong acid or oxidative solutions destructed the microporous structures and changed the size uniformity of fibers in the BCNMs, resulting in a decline in formaldehyde adsorption capacity. A synergistically physical–chemical adsorption took place during formaldehyde adsorption by the modified biomass nanofiber membranes, due to the coexistence of suitable functional groups and porous structures in the membranes.

## 1. Introduction

Improvements in living standards have led to a growing demand for high-quality indoor air. However, furniture, flooring, wardrobes, ceilings, wallpapers, and other decorative materials made from wood composites with formaldehyde resin-based adhesives can emit irritating formaldehyde gas. Given that individuals spend around 80 percent of their time indoors, maintaining high indoor air quality is vital for protecting human health. Formaldehyde, a colorless gas with a pungent odor, has been classified as a Group I teratogen and carcinogen by international authoritative organizations [1,2,3]. Short-term exposure to a formaldehyde-rich environment can lead to symptoms such as chest distress, heart palpitations, and headaches [4]. In contrast, long-term exposure significantly increases the risk of cancers, especially leukemia [5,6]. Therefore, it is imperative to develop a rapid and efficient method for removing formaldehyde.

Several technologies, including chemical reaction methods, photocatalytic methods, plant purification, and adsorption techniques, have been utilized to tackle gaseous formaldehyde pollution [7,8]. Among them, adsorption is the most cost-effective, user-friendly, adaptable, and readily available option, with significant potential to effectively address formaldehyde pollution, especially at low concentrations [9]. Common adsorbents like activated carbon, silica gel, and zeolite often show suboptimal adsorption performance. Porous nanofiber membranes produced through electrospinning exhibit remarkable formaldehyde adsorption capabilities, which can be further improved through carbonization and plasma treatment. Biomass-derived adsorbents with abundant feedstocks, low costs, renewability, environmental friendliness, and excellent adsorption performance have attracted increasing attention in recent years. Therefore, there is promising potential for developing biomass nanofiber membranes as formaldehyde adsorbents.

The key factors for effective formaldehyde adsorption are the porosity and the available functional groups [10,11]. Biochar and activated carbon, made from the carbonization of wood, bamboo, straw, husks, shells, and peels, demonstrate good adsorption performance for different pollutants [12,13,14]. The pore structure of biomass nanofiber membranes can be improved by carbonization treatment, thereby promoting their physical adsorption performance. A zeolitic imidazolate framework (ZIF-8)-based carbon nanofiber membrane was fabricated through an in situ solvothermal process and carbonization by Sun et al. [15]. Compared with the non-carbonized nanofiber membranes, the carbonized one achieved a higher adsorption capacity.

Cold plasma treatment provides a simple and sustainable method for introducing functional groups into carbon materials without destroying their structural integrity [16]. Our previous studies indicate that carboxyl groups and pyrrole nitrogen can be effectively integrated onto biomass micro/nanofibers through cold plasma activation, which enhances formaldehyde adsorption by altering the electrostatic potential distribution and facilitating the formation of hydrogen bonds, Lewis acid-base interactions, and van der Waals forces [17]. On the other hand, wet chemical treatment with zinc chloride, potassium permanganate, nitric acid, sulfuric acid, and thiourea can significantly improve the physical and chemical properties of adsorbents, thereby increasing their formaldehyde adsorption capacity [18,19,20,21]. Sulfur and nitrogen groups were introduced into activated carbon using thiourea by Unglaube et al. [22], improving formaldehyde adsorption through specific interactions with positively charged carbon atoms. The ultrafine pores of carbon-fiber fabrics were significantly increased by impregnation with urea, thiourea, dicyandiamide, or penicillin by De Falco G et al. [23], resulting in an increase in formaldehyde adsorption performance. The adsorption behavior can be enhanced by chemical interactions between formaldehyde and functional groups such as pyridine, amine, and sulfone/sulfonic acid. Furthermore, the abundant O- and N-containing functional groups on the surface of the adsorbents reinforced the polar interactions. The levels of functional groups, especially for amino groups and phenolic hydroxyl, were increased on bamboo activated carbon by ammonium acetate, greatly enhancing its chemisorption capacity for formaldehyde [24].

Our previous works have demonstrated that bamboo-based carbon microfibers modified by carbonization and plasma, as well as microcrystalline cellulose (MCC)/chitosan (CS) nanofibers modified only by plasma, can effectively remove gaseous formaldehyde [17,25,26,27]. In this study, we conducted a further comparative study on the absorption of gaseous formaldehyde by electrospun porous MCC/CS nanofiber membranes modified by combining carbonization with oxygen plasma activation and chemical treatment with nitric acid, sulfuric acid, hydrogen peroxide, and urea. The changes in the micro-morphology, functional groups, crystallinity, and surface composition of various samples, along with their formaldehyde adsorption performance and kinetic fitting, were characterized and comparatively analyzed.

## 2. Results and Discussion

### 2.1. Analysis of Morphology of BCNMs Before and After Chemical Treatment

**Figure 1:**
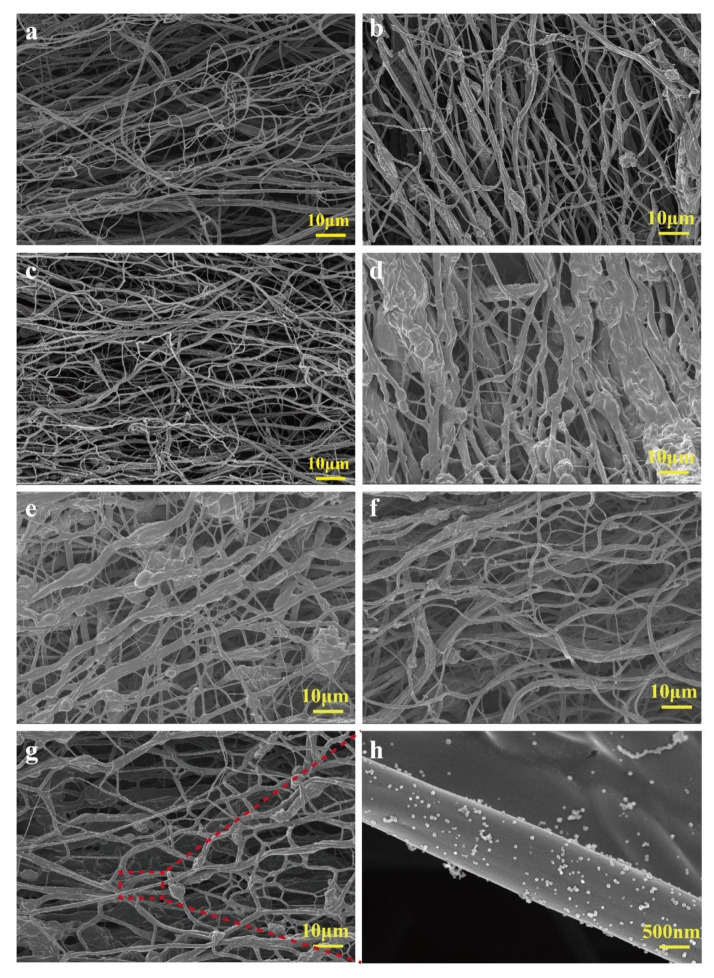
Micro-morphology of biomass nanofiber membranes: (**a**) MCC/CS; (**b**) BCNM; (**c**) BCNM-O_2_; (**d**) BCNM-H_2_O_2_; (**e**) BCNM-H_2_SO_4_; (**f**) BCNM-HNO_3_; (**g**,**h**) BCNM-urea.

Figure 1 shows the variations in micro-morphologies for MCC/CS nanofibrous membranes modified by combining carbonization with O_2_ plasma activation or chemical treatment with various solutions. The pristine MCC/CS nanofibrous membrane displays uniform and porous network structures, with fiber diameters of less than 1 μm (Figure 1a). Both BCNM and BCNM-O_2_ exhibited a uniform fiber distribution with a smooth surface and partial fiber fractures when they were carbonized at 800 °C and subsequently activated in an O_2_ plasma atmosphere (Figure 1b,c). The arrangement of carbon nanofiber membranes without chemical treatment showed a trend of fiber contraction, enhancing the stability of the membrane structure. A few nodes and aggregations were observed, and the macropores derived from the network structure were relatively uniform, as illustrated in Figure 1a–c, where the dispersion and uniformity of the fibers treated in an oxygen plasma atmosphere tended to be improved.

However, the micro-morphology of the BCNMs was obviously changed after impregnation with H_2_O_2_, as shown in Figure 1d. The membrane exhibited significant agglomeration, with these fibers clustering together to form larger-diameter rods. This agglomeration disrupted the initially uniform distribution of the fibers, resulting in only a small fraction of the fibers retaining their original morphology. This suggests that the initial porous network structure of the fiber membrane was difficult to maintain under the strongly oxidizing H_2_O_2_ [28]. The BCNMs modified by H_2_SO_4_ presented an increasing fiber diameter with thicker nodes, as illustrated in Figure 1e. The overall network structure of the fibers remained relatively stable, although the fiber distribution became uneven, which can be explained by prolonged exposure to a high concentration of the H_2_SO_4_ solution, leading to fiber decomposition and merging, with a coarser morphology. In contrast, the uniform arrangement of fibers tended to be curved and thin, with some entanglement, instead of the nodules or agglomeration after modification with HNO_3_ (Figure 1f). The morphology of the fiber membrane after impregnation with urea solution is illustrated in Figure 1g,h; there was little change in the fiber shape compared to the control. Nevertheless, a significant accumulation of urea particles on individual fibers was noticed, as shown in Figure 1h, which was due to the evaporation of water during the impregnation and drying processes, facilitating the particles’ reattachment. The pores on the fiber surface can be blocked by these urea particles, resulting in a reduction in the available adsorption sites for capturing formaldehyde molecules [29].

### 2.2. Fourier-Transform Infrared Spectroscopy (FTIR) and X-Ray Diffraction (XRD) Analysis

**Figure 2:**
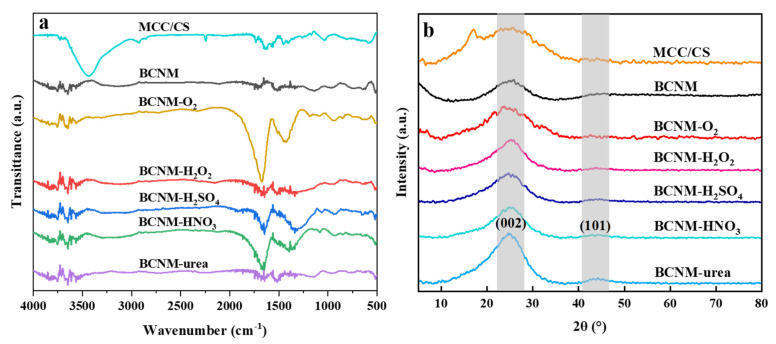
(**a**) FTIR and (**b**) XRD of biomass nanofiber membranes.

The changes in functional groups and crystallinity for biomass nanofiber membranes are shown in Figure 2. As illustrated in Figure 2a, the peaks in the range of 3750–3250 cm^−^^1^ are associated with the stretching and bending vibrations of the -OH and -NH in MCC and CS [30,31]. The strong adsorption bands are prominently observed in the range of 1600–1700 cm^−1^, due to the stretching vibrations associated with the C=O bonds in carbonyl groups [32]. The corresponding peaks in the BCNM were relatively weak, indicating that a great number of O-containing functional groups decomposed during the carbonization process. After chemical treatment, particularly with H_2_SO_4_ and HNO_3_, the intensity of the C=O peak significantly increased, implying that the treatment effectively introduced O-containing functional groups onto the surface of the carbon nanofiber membranes. The BCNM-O_2_ exhibited a higher density for the C=O peak compared to the chemically impregnated BCNMs, which indicated that the O_2_ plasma treatment introduced more O-containing functional groups. The absorption peaks at 1560–1480 cm^−^^1^ were related to the stretching and bending vibrations of -NH_2_ in chitosan and -CH_2_ in PAN [31]. There were no significant variations in the absorption bands of MCC/CS, BCNM, BCNM-H_2_O_2_, and BCNM-urea. However, they disappeared and shifted to a broad band ranging from 1495 to 1112cm^−1^ in the BCNM-O_2_, BCNM-H_2_SO_4_, and BCNM-HNO_3_, corresponding to the stretching vibrations of C-O bonds in oxidized carbon and in acid, alcohol, phenol, ether, and/or ester groups [33]. This also implies that O-containing functional groups were introduced. The multiple peaks around 1100–560 cm^−1^ were assigned to the out-of-plane bending vibrations of C-C, C-N, or -CH bonds.

It can be seen in Figure 2b that the diffraction peak at 2θ = 17° in MCC/CS corresponds to the overlapping peaks of II-type crystals in chitosan and the (110) crystal face of cellulose. Its disappearance after carbonization indicated that the crystal structures of chitosan and cellulose were transformed to the graphite-like carbon nanofiber membranes. Two distinct crystal planes were clearly observed in the BCNM: One showed a prominent peak at nearly 24°, corresponding to the (002) diffraction plane of graphite carbon. The shape of BCNM-O_2_ was similar to that of BCNM, with the peak width becoming slightly narrower after treatment with four chemical solutions. Another weak shoulder peak emerged at 44°, corresponding to the (101) crystal plane of graphite [34,35]. There was no significant change in its position or intensity after O_2_ plasma activation and immersion treatment with different chemical solutions. This indicates that O_2_ plasma activation and chemical treatment can alter the chemical properties on the surface of carbon nanofiber membranes but hardly affect the crystal structure and network architecture of the membranes.

### 2.3. X-Ray Photoelectron Spectroscopy (XPS) Analysis

**Figure 3:**
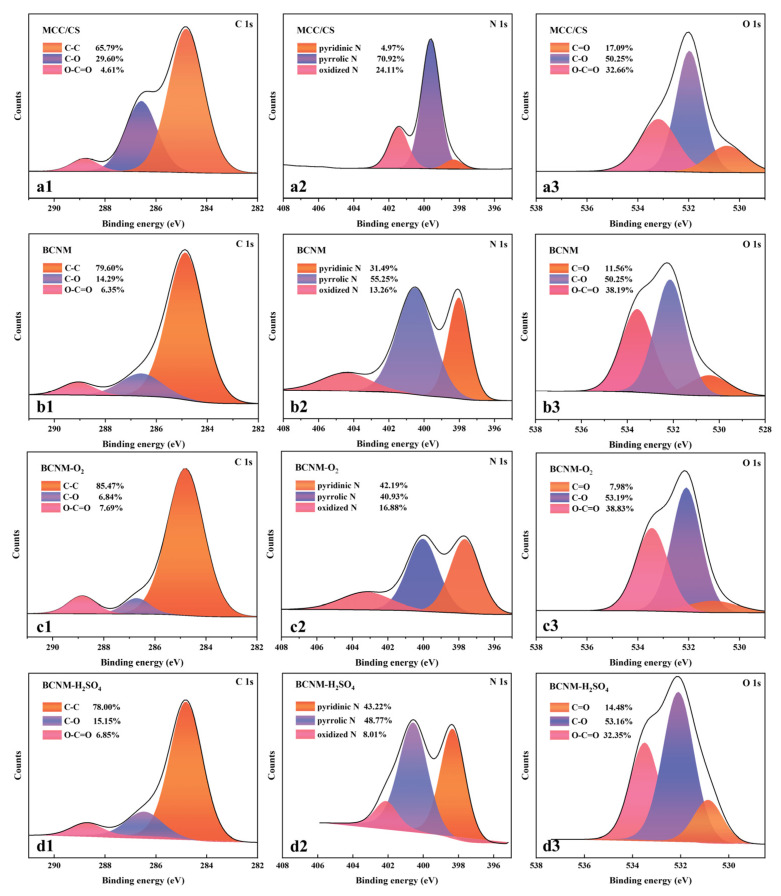
XPS spectra of C 1s, N 1s, and O 1s regions: (**a1**–**a3**) MCC/CS; (**b1**–**b3**) BCNM; (**c1–c3**) BCNM-O_2_; (**d1**–**d3**) BCNM-H_2_SO_4_.

The atomic states and chemical composition on the surface of various samples were further analyzed, as shown in Figure 3, Figures S1 and S2. The peak intensities of O 1s and N 1s in MCC/CS were significantly decreased after carbonization, indicating the decomposition of O- and N-containing functional groups in the BCNM. The peak intensities of N 1s were enhanced for the BCNMs modified by impregnation with HNO_3_ and urea (Figure S2). The peak intensities of O 1s for the BCNMs were also increased by O_2_ plasma activation, as well as H_2_O_2_ and H_2_SO_4_ immersion (Figure 3 and Figure S2). The changes in functional group contents suggest that specific functional groups can be effectively introduced onto the surface of BCNMs through O_2_ plasma activation and chemical modifications [36]. Among the samples modified by chemical treatment, the urea-modified BCNMs displayed the highest content of N-containing functional groups, whereas the H_2_SO_4_-modified BCNMs showed the highest content of O-containing functional groups. This variation is consistent with their formaldehyde adsorption capacities, highlighting a strong correlation between the functional group content and formaldehyde adsorption performance [37].

The detailed binding energies and surface element distributions of different biomass nanofiber membranes are listed in Table 1 and Table S1. The high-resolution C 1s spectra for all of the samples exhibit three peaks, corresponding to C-C (284.8 eV), C-O (285.3 eV), and O-C=O (289.1 eV) [38,39]. The intensities of the C-O peaks decreased, while the levels of the O-C=O peaks increased, after the BCMFs were treated by O_2_ plasma activation and chemical immersion, as shown in Figure 3(a1–d1) and Figure S1(a1–c1). The N 1s spectra for all samples present three distinct peaks, corresponding to pyridinic N (398.2 eV), pyrrolic N (399.9 eV), and oxidized N (402.5 eV) [40]. The contents of pyridinic N, pyrrolic N, and graphitic N in BCNM-HNO_3_ and BCNM-urea increased to 3.12, 4.95, and 0.80% and to 7.47, 5.65, and 1.15%, respectively, from 2.20, 3.85, and 0.92% for BCNM. It was proven that the levels of N-containing functional groups in the BCNM were effectively elevated after chemical treatment in HNO_3_ and urea solutions. The deconvolution of the O1s spectra shows three major peaks, corresponding to C-O (532.1 eV), C=O (531.0 eV), and O-C=O (533.2 eV) [17]. The contents of these three O-containing functional groups in BCNM-H_2_O_2_, BCNM-H_2_SO_4_, and BCNM-O_2_ increased to 0.73, 7.83, and 6.55%, 2.02, 7.40, and 4.51%, and 1.28, 8.83, and 6.22%, respectively, from 1.15, 5.04, and 3.82% for the BCNM. Notably, the amount of carboxyl groups significantly rose.

The organic element contents of different BCNMs are presented in Table 2. The (O+N)/C ratio serves as an indicator of hydrophilicity, with a high value reflecting great hydrophilicity [23]. The (O+N)/C ratio of MCC/CS reduced from 1.0974 to 0.6976 after carbonization, which is in accordance with the decomposition of polar functional groups (OH, C-O-C, and NH_2_), as verified by the FTIR and XPS analyses. The (O+N)/C value of BCNM-O_2_ increased to 0.9389, approaching that of MCC/CS before carbonization. Meanwhile, the N content of BCNM-O_2_ was elevated as well. This indicates that the O_2_ plasma activation was a more effective method for enhancing the polarity and O-containing functional groups of the BCNM compared with chemical treatment. Additionally, the O_2_ plasma treatment reduced the amount of carbon and increased the relative nitrogen content. Among the four chemical treatments, BCNM-H_2_SO_4_ displayed the highest (O+N)/C value (0.7763), but the lowest contents of N and O and the (O+N)/C value were observed in BCNM-H_2_O_2_. The N and O contents of both BCNM-O_2_ and BCNM-H_2_SO_4_ were higher than those of the BCNM, demonstrating that impregnation treatment with H_2_SO_4_ solution was effective to enhance the functional group contents. However, it is necessary to control the concentration and immersion time within a certain range to ensure the effective enhancement of element contents without causing damage to the original material structure [41].

### 2.4. Analysis of Adsorption Properties of Formaldehyde

#### 2.4.1. Analysis of Adsorption Kinetics

The adsorption mechanism of formaldehyde by biomass nanofiber membranes was further investigated through the fitting of classical kinetics models. The pseudo-first-order and pseudo-second-order kinetic models based on Equations (1) and (2) were fitted [42].

(1) $$q_{t}=q_{e}(1-e^{{-tk}_{1}})$$

(2) $$q_{t}=\frac{q_{e}t}{\frac{1}{k_{2}q_{e}}+t}$$

where q_t_ and q_e_ signify the adsorption capacity (mg g^−^^1^) at the specific time t (h) and at equilibrium, respectively. The constants (k_1_ and k_2_) represent the rate constants for the respective models, expressed in h^−^^1^ and g mg^−^^1^ h^−^^1^, respectively.

The Elovich model and the intraparticle diffusion model are represented by Equations (3) and (4), respectively:

(3) $$q_{t}=\frac{1}{\beta }\ln(\alpha \beta t+1)$$

(4) $$q_{t}=k_{f}t^{\frac{1}{2}}+C$$

where q_t_ signifies the adsorption capacity of formaldehyde at a specific time (mg g^−1^), α represents the adsorption rate at the beginning stage (mg g^−1^ min^−1^), β refers to the desorption constant (g mg^−1^) [43], and k_f_ and C stand for the diffusion constant (mg g^−1^ min^−1/2^) and the constant of boundary layer thickness, respectively [44].

**Figure 4:**
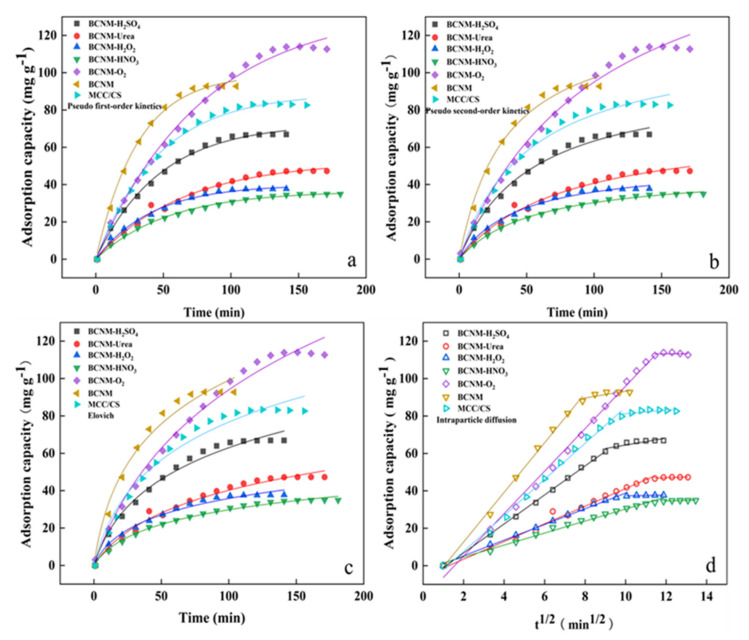
Kinetics models for formaldehyde adsorption by biomass nanofiber membranes, (**a**) the pseudo-first-order; (**b**) pseudo-second-order; (**c**) Elovich; (**d**) intraparticle diffusion kinetic models.

As depicted in Figure 4, an analysis was performed comparing several dynamic models. The specific parameters and correlation coefficients (R^2^) for these models are also shown in Table 3. The R^2^ values pertaining to the pseudo-first-order and pseudo-second-order kinetics models range from 0.9904 to 0.9962 and from 0.9866 to 0.9968, respectively, showing better fitting effects than those of the Elovich model, whose R^2^ values range from 0.9743 to 0.9921. This suggests that a synergistic relationship was observed between physical and chemical adsorption throughout the formaldehyde adsorption process of the biomass nanofiber membranes [45,46]. The Elovich kinetic model exhibited a relatively high R^2^ value as well, indicating the presence of heterogeneous surface chemical adsorption.

In addition, the curve of q_t_ and t^1/2^ did not intersect at the original point, implying that intraparticle diffusion was not the only factor influencing the adsorption performance. The adsorption capacity of nanofiber membranes may be affected by other mass transfer factors [47]. Additionally, the curve for the fitting model of intraparticle diffusion can be divided into two stages, due to the occurrence of diffusion of formaldehyde molecules into the pores. In the initial stage, the curve’s slope was higher than that in the second stage, corresponding to a higher adsorption capacity, which was attributed to the abundant macropores and mesopores in the biomass nanofiber membranes, which can offer numerous adsorption sites and channels for the physical capture of formaldehyde. On the other hand, the decrease in slope during the second stage indicated a slower rate of adsorption in the micropores, which was attributed to resistance to mass transfer, changes in void distribution, and pore size [48].

#### 2.4.2. Formaldehyde Adsorption Performance

**Figure 5:**
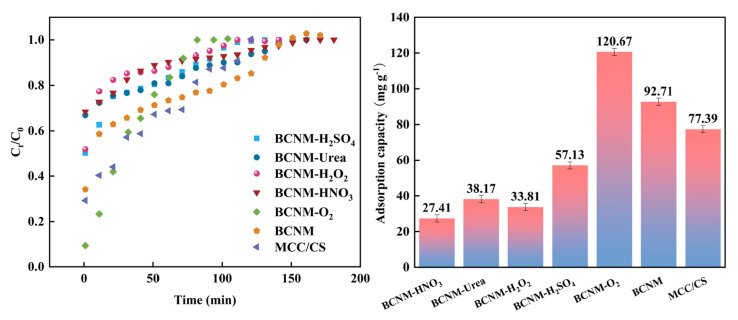
The breakthrough curves and formaldehyde adsorption capacities of biomass nanofiber membranes.

Figure 5 illustrates the breakthrough curves and adsorption performance of BCNM for gaseous formaldehyde under different modifications. Based on the breakthrough curve shown in Figure 5, the dynamic data and corresponding adsorption capacity under saturated conditions were determined. In the initial adsorption phase, a substantial number of formaldehyde molecules were absorbed onto the pores and vacant sites of functional groups in the sample, leading to a rapid decrease in formaldehyde concentration. As the accessible adsorption sites diminished, the rate of decline in formaldehyde concentration slowed, indicating that saturation was approaching [47]. The various biomass nanofiber membranes’ adsorption capacity for gaseous formaldehyde was ranked as follows: BCNM-O_2_ > BCNM > MCC/CS > BCNM-H_2_SO_4_ > BCNM-urea > BCNM-H_2_O_2_ > BCNM-HNO_3_. Breakthrough curve analysis revealed that the chemically modified BCNM reached adsorption equilibrium at a faster rate than the control. In contrast, the adsorption equilibrium time of BCNM-O_2_ was notably longer than that of the BCNM. This was due to an increase in effective functional groups and chemical active sites for formaldehyde molecule adsorption after O_2_ plasma modification. This conclusion is consistent with the findings from the earlier SEM analysis.

According to the adsorption characteristics of gaseous formaldehyde, N- and O-containing functional groups, including pyridine-N, pyrrole-N, and carboxyl, were key factors in enhancing the adsorption performance [49]. Thus, the optimal formaldehyde adsorption capacity was achieved at 120.67 mg g^−1^ for BCNM-O_2_, whose surface and interior had the highest oxygen content (16.03%) and a relatively high N content (14.22%), as shown in Table 1 and Table 2. Despite a significant increase in the amounts of functional groups on the surface of the chemically treated membranes, the adsorption capacity was not correspondingly improved. It can be speculated that after immersion in strong acidic or alkaline solutions, the nano-network structure of the BCNMs experienced significant alterations, resulting in the destruction of the microporous structure and uneven fiber thickness. These changes may be the primary reason for the reduced formaldehyde adsorption performance. Therefore, the sufficient channels and pores of BCNMs should be maintained and generated with the introduction of available O- and N-containing functional groups on their inner and outer surface for the highly efficient adsorption of formaldehyde molecules through two key steps: “rapid entry and efficient capture” [50].

## 3. Materials and Methods

### 3.1. Materials

CS with a deacetylation degree of 95% and MCC particles were purchased from Sinopharm Chemical Reagent Co., Ltd. (Shanghai, China). Hydrogen peroxide (H_2_O_2_), nitric acid (HNO_3_), and dimethylformamide (DMF) were supplied by Tianjin Fengchuan Chemical Reagent Technology Co., Ltd. Sulfuric acid (H_2_SO_4_) and urea were obtained from Yunnan Shandian Reagent Co., Ltd. 1-Ethyl-3-methylimidazole acetate was obtained from our lab.

### 3.2. Preparation and Modification of Biomass Carbon Nanofiber Membranes

The electrospinning process was performed using the ES-BS200 (Zhongqing Kejing Technology, Xiamen, China) instrument to prepare MCC/CS nanofibrous membranes based on previous technological parameters, including weight ratio, voltage, injection rate and collection distance [26]. Subsequently, the obtained MCC/CS nanofibrous membranes were dried at 40 °C under vacuum conditions and then pre-oxidized and carbonized in a nitrogen atmosphere at 800 °C for 2 h to form porous biomass carbon nanofiber membranes (BCNMs). The obtained BCNMs were then subjected to modification in a cold plasma apparatus (VP-RS8, Sunjune, China) at 60 W for 1 min under an O_2_ atmosphere, and through wet chemical treatments, corresponding to a 10% urea solution, a 30% HNO_3_ solution, a 30% H_2_O_2_ solution, and a 2N H_2_SO_4_ solution. The BCNMs were fully immersed in four solutions for 3 h. Subsequently, the samples were extracted and rinsed five times with distilled water, followed by two washes with ethanol. After treatment, they were dried to a constant weight in a vacuum-drying oven at 60 °C.

### 3.3. Characterization and Measurement

Micro-morphological observations of the BCNMs were conducted using a scanning electron microscope (SEM, ZEISS Gemini 300, Jena, Germany) operated at 3 kV. The FTIR spectra for the samples were obtained within the range of 4000–400 cm^−1^, utilizing an IS5 Nicolet instrument (Thermo Fisher Scientific, Waltham, MA, USA). X-ray diffraction (XRD, IV Ultima, Rigaku, Japan) with a Cu Kα radiation source (λ = 0.1542) was utilized to investigate the crystalline structures of BCNMs, measuring Bragg’s angle from 5 to 80° at a step increment of 0.02° and voltage of 40 kV. X-ray photoelectron spectroscopy (XPS, K-Alpha, Thermo Scientific, Waltham, MA, USA) was employed with monochromatic Al-Kα radiation and a step increment of 0.1 eV to characterize the electronic states of the surface elemental components of the BCNMs. The gaseous HCHO adsorption device and testing method were those used in our previous report [27]. The adsorption capacity of HCHO was calculated using Equation (5):

(5) $$Q_{t}=\frac{Q_{f}C_{\mathrm{in}}}{m}\int_{0}^{t}\left(1-\frac{C_{\mathrm{out}}}{C_{\mathrm{in}}}\right)dt$$

where m represents the weight of the samples (g), Q_f_ is the inlet flow rate (L min^−1^), and C_in_ and C_out_ represent the HCHO concentrations at the inlet and outlet, respectively (mg L^−1^).

## 4. Conclusions

This study explored the adsorption efficiency of gaseous formaldehyde pollutants by biomass nanofiber membranes modified through carbonization and subsequent modifications, including cold plasma activation (O_2_) and chemical treatment. Different O- and N-containing functional groups were successfully introduced onto the surface of the BCNMs. BCNM-O_2_ showed the optimal formaldehyde absorption capacity (120.67 mg g^−1^), which was attributed to its highest content of N- and O-containing functional groups. However, chemical treatment in strong acidic or oxidative solutions destructed the microporous structures and changed the size uniformity of the fibers, with alterations in the nano-network structure of the BCNMs, resulting in a decline in formaldehyde adsorption capacity. A synergistically physical and chemical adsorption took place for the modified biomass nanofiber membranes, due to the coexistence of suitable functional groups and porous structures in the membranes.

## Figures and Tables

**Table 1:** Binding energy and surface element composition of various biomass nanofiber membranes.

Atomic States and Contents	MCC/CS	BCNM	BCNM-O_2_	BCNM-H_2_SO_4_
N 1s content (%)	8.31	7.16	2.06	6.23
Pyridinic N (%)	0.41	2.20	0.87	2.69
Binding energy (eV)	398.31	398.01	397.65	398.39
Pyrrolic N (%)	5.89	3.85	0.84	3.04
Binding energy (eV)	399.61	400.49	400.02	400.53
Oxidized N (%)	2.00	0.92	0.35	0.50
Binding energy (eV)	401.44	404.41	403.22	402.99
O 1s content (%)	13.92	10.27	16.03	13.93
C=O (%)	2.38	1.15	1.28	2.02
Binding energy (eV)	530.48	530.42	531.02	530.69
C–O (%)	6.99	5.04	8.83	7.40
Binding energy (eV)	531.96	532.13	532.08	532.09
O–C=O (%)	4.55	3.82	6.22	4.51
Binding energy (eV)	533.18	533.56	533.44	533.75

**Table 2:** Organic element composition of biomass nanofiber membranes.

Groups	C/%	N/%	H/%	O/%	(O+N)/C
MCC/CS	44.63	4.04	6.39	44.94	1.0974
BCNM	57.97	9.30	1.535	31.14	0.6976
BCNM-O_2_	51.34	14.22	0.886	33.467	0.9389
BCNM-H_2_O_2_	59.57	11.05	0.642	28.623	0.6660
BCNM-H_2_SO_4_	54.88	11.39	0.768	31.213	0.7763
BCNM-HNO_3_	58.28	11.99	0.516	29.104	0.7051
BCNM-Urea	57.86	14.37	0.764	26.912	0.7135

**Table 3:** Kinetic parameters for different models.

Sample	Pseudo-First-Order			Pseudo-Second-Order			Elovich		
	q_e_ (mg g^−1^)	k_1_ (min^−1^)	R^2^	q_e_ (mg g^−1^)	k_2_ (g mg^−1^ min^−1^)	R^2^	α (mg g^−1^ min^−1^)	β (g mg^−1^)	R^2^
BCNM-H_2_SO_4_	73.8122	0.022	0.9955	100.9800	0.00021	0.9909	2.5454	0.0378	0.9840
BCNM-Urea	53.7144	0.0164	0.9904	75.5500	0.00022	0.9871	1.2293	0.0473	0.9817
BCNM-H_2_O_2_	40.5122	0.0252	0.9936	53.4455	0.00045	0.9906	1.8001	0.0754	0.9838
BCNM-HNO_3_	36.8838	0.0193	0.9962	48.1804	0.00032	0.9968	1.2111	0.0794	0.9921
BCNM-O_2_	134.1152	0.0126	0.9943	193.3992	0.00004	0.9913	2.1183	0.0154	0.9878
BCNM	99.2777	0.0320	0.9952	130.5984	0.00022	0.9866	4.9319	0.0267	0.9745
MCC/CS	88.9330	0.0215	0.9956	117.5722	0.00016	0.9867	2.8947	0.0293	0.9743

## Data Availability

The data presented in this study are available upon request from the corresponding author.

## Supplementary Materials

The following supporting information can be downloaded at https://www.mdpi.com/article/10.3390/molecules30102184/s1: Figure S1: XPS spectra of biomass nanofiber membranes; Figure S2: C 1s, N 1s, and O 1s energy spectra of (a) BCNM-H_2_O_2_, (b) BCNM-HNO_3_, and (c) BCNM-urea; Table S1: Binding energy (eV) and surface element distribution (%) of BCNM-H_2_O_2_, BCNM-HNO_3_, and BCNM-urea.
