# Supplementary material for: A Comparative Study on Absorption of Gaseous Formaldehyde by Electrospun Biomass Carbon Nanofiber Membranes Modified by Plasma Activation and Chemical Treatment
Source: Molecules. 2025 May 16;30(10):2184. doi: 10.3390/molecules30102184 (PMC12113733; doi:10.3390/molecules30102184)
Supplement: Supplementary file 1 [file molecules-30-02184-s001.zip › molecules-3512456-supplementary.pdf]

Support information

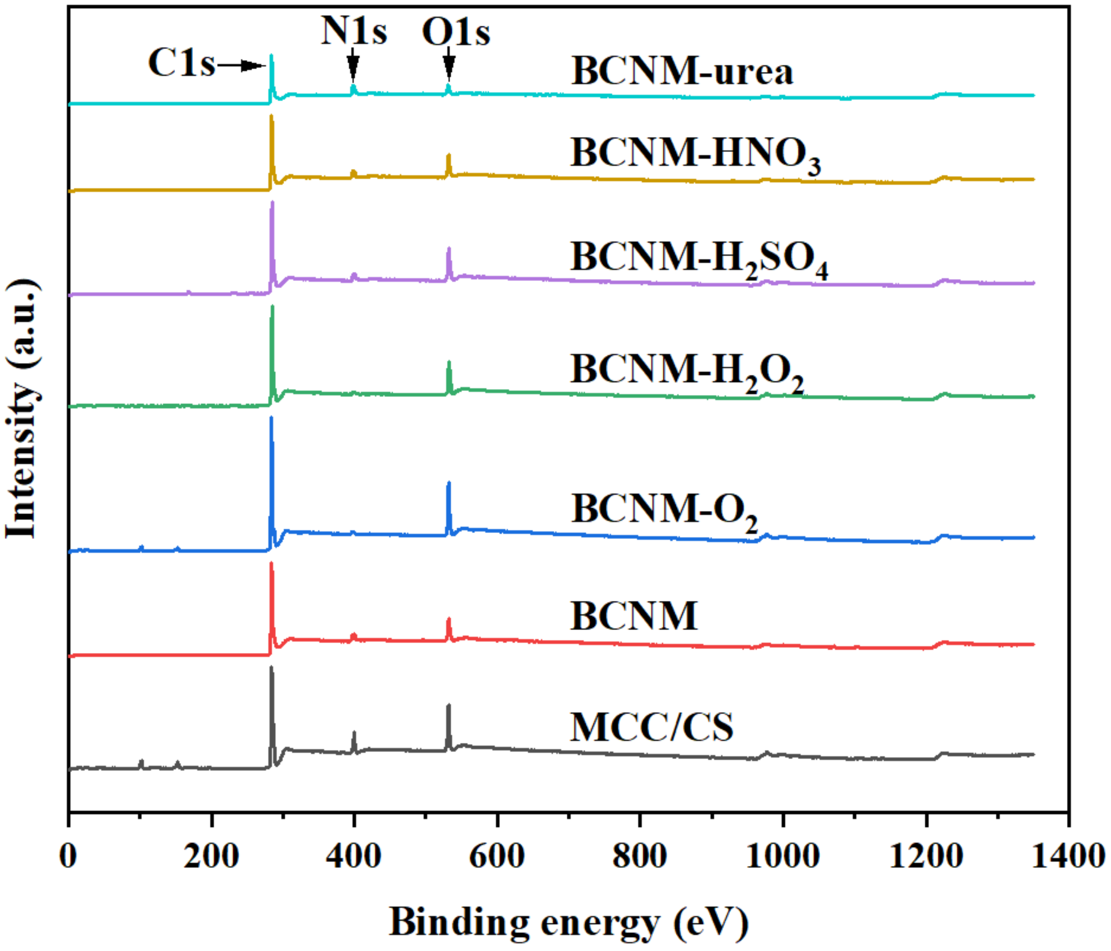

Figure S1. XPS spectra of biomass nanofiber membranes.

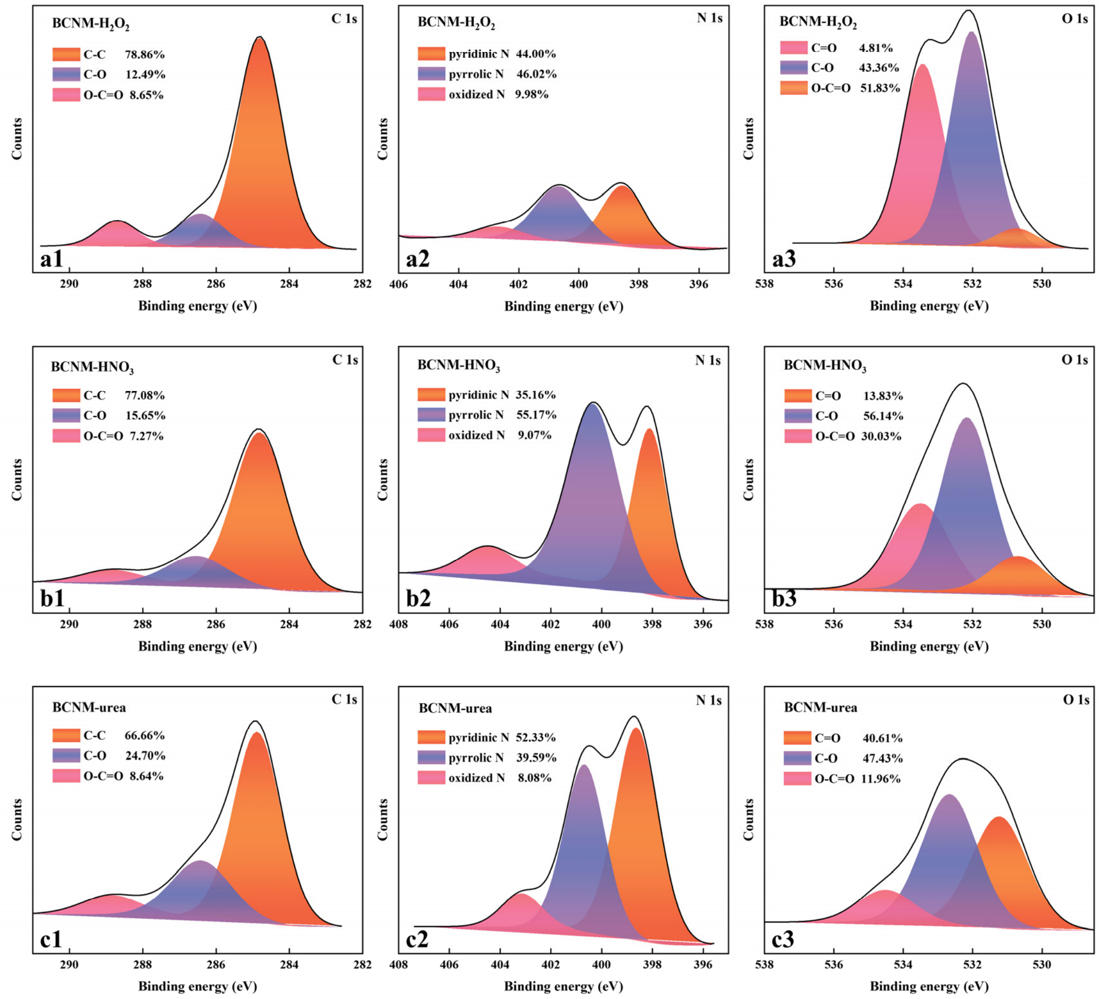

Figure S2. C 1s, N 1s, and O 1s energy spectra of (a) BCNM-H_2O_2; (b) BCNM-HNO_3; and (c) BCNM-urea.

Table. S1 Binding energy (eV) and surface element distribution (%) of BCNM-H_2O_2, BCNM-HNO_3, and BCNM-urea

sample	BCNM-H_2O_2	BCNM-HNO_3	BCNM-urea
N 1s content (%)	2.48	8.87	14.27
Pyridinic N (%)	1.09	3.12	7.47
Binding energy (eV)	398.32	398.30	397.74
Pyrrolic N (%)	1.14	4.95	5.65
Binding energy (eV)	400.43	400.40	399.69
Oxidized N (%)	0.25	0.80	1.15
Binding energy (eV)	402.40	402.66	401.89
O 1s content (%)	15.11	11.66	7.88
C=O (%)	0.73	1.61	1.87
Binding energy (eV)	530.73	530.69	530.52
C–O (%)	7.83	6.55	5.02
Binding energy (eV)	532.15	532.03	532.04
O–C=O (%)	6.55	3.50	0.99
Binding energy (eV)	533.79	533.70	533.51
